# Use of D‐optimal combined design methodology to describe the effect of extraction parameters on the production of quinoa–barley malt extract by superheated water extraction

**DOI:** 10.1002/fsn3.2184

**Published:** 2021-02-18

**Authors:** Samireh Sabah, Anoshe Sharifan, Afshin Akhonzadeh Basti, Behrooz Jannat, Maryam TajAbadi Ebrahimi

**Affiliations:** ^1^ Department of Food Science and Technology, Science and Research Branch Islamic Azad University Tehran Iran; ^2^ Department of Food Hygiene Faculty of Veterinary Medicine University of Tehran Tehran Iran; ^3^ Halal Research Center Islamic Republic of Iran Tehran Iran; ^4^ Department of Biology Central Tehran Branch Islamic Azad University Tehran Iran

**Keywords:** barley malt, combined design, extraction, quinoa, superheated water extraction

## Abstract

Superheated water extraction was applied to produce quinoa–barley malt extract. D‐optimal combined design was used to optimize the extraction conditions (time (min), solid–water ratio and particle size to obtain maximum protein and carbohydrate content, and minimum turbidity and pH. Quinoa flour (10%–30%), barley malt flour (70%–90%), different particle sizes (*F* = 420 µm, *G* = 710 µm), time (15–45 min), and solid–water ratio (0.1–0.2) were selected as independent variable and protein, carbohydrate, turbidity, and pH as dependent factors. Polynomials models satisfactorily fitted the experimental data with the *R* ^2^ values of .9961, .9909, .9949, and .9987, respectively. The protein and carbohydrate value was affected by superheated water extraction parameters. Our results revealed that increasing quinoa/barley malt ratio has significant effect on the turbidity and pH. The optimum extraction conditions were quinoa flour (30%), barley malt flour (70%), solid–water ratio (0.2), time (45 min), and particle size (*F* = 420 µm).

## Graphical abstract

We develop a combine design methodology for extraction of valuble material from quina–malt mix by subcritical water extraction, as a new extraction method. The extraction process is based on the use of subcritical water that as an environmentally friendly method has applied to prepare the quina–malt mix extract for using in functional beverage.

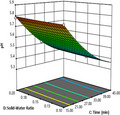

## 1 INTRODUCTION

Quinoa **(** *Chenopodium quinoa* Willd.) plant that belongs to the *Chenopodiaceae* family is a pseudocereal and the history of its human consumption reaches back 7,000 years (Abugoch et al., 2009; Guerreo‐Ochoa et al., 2015). These seeds have encouraged FAO to determine potential cultivation areas in Europe, Asia, Africa, Australia, and North America in order to expand its cultivation to different geographical regions, and it is also considered an option to solve human nutrition problems (Guerreo‐Ochoa et al., 2015). Because of its high nutritive potential and genetic diversity, quinoa is classified by FAO as one of humanity's promising crops that can contribute to food security in the twenty‐first century (Bazile et al., 2015; Guerreo‐Ochoa et al., 2015). Moreover, FAO has officially declared the year 2013 as the ‘International Year of the Quinoa’ (Bazile et al., 2015).

Quinoa is a good source of macro‐ and micronutrients, with protein contents comparable to conventional cereals and excellent nutritional properties connected to the high quality of the protein. Pseudocereals are also particularly good sources of minerals such as iron, zinc, calcium, magnesium, manganese, and copper content that is higher than conventional cereals (Nisar et al., 2017; Pereira et al., 2019). Quinoa is a gluten‐free seed which is suitable for celiac patients as well as people who have wheat allergy. It has benefit for high‐risk group consumers such as children, the elderly, women prone to osteoporosis, people with anemia, diabetes, dyslipidemia, and obesity (López et al., 2018; Navruz‐Varli & Sanlier, 2016).

Barley ranks fourth in quantity produced and cultivation area of cereals in the world and has a long history of use as a source of human nutrition. Clinical studies have shown that the intake of β‐glucan from either barley flour or barley malt products can control cardiovascular disease (CVD), type 2 diabetes, obesity, colorectal cancer, and lower blood glucose levels in humans (Arendt & Zannini, 2013; Chappell et al., 2017; De Arcangelis et al., 2019; Suriano et al., 2018).

There are different methods for extracting the functional compounds from cereal or pseudocereal, but most of them were traditionally extracted by organic solvents such as methanol, ethanol, and acetone. However, conventional methods are time‐consuming and some of the solvents are toxic. Recently, advanced extraction technologies such as ultrasound, supercritical CO2, microwave, and superheated water extraction (SWE) have been developed to overcome these problems.

Subcritical water extraction or superheated water extraction (SWE) has become a popular green extraction technique for the isolation of different classes of compounds from natural matrices. Low price, safety and green character of water, good yields of target compounds, and reduced energy consumption make this technique favorable for potential industrial applications (Nastić et al., 2018).

SWE technique has been studied in recent years to extract functional compounds from diverse matrix of food by‐products and agricultural products (Erşan et al., 2018; Miró‐Abella et al., 2018), such as bagasse waste, potato peel, wheat straw, tea industry by‐products (Naffati et al., 2017), ginger extract (Švarc‐Gajić et al., 2017), soybean products (Moras et al., 2017), traditional Serbian medicinal plants (Nastić et al., 2018), and Pacific oyster (Getachew et al., 2019).

There have been statistical and soft‐computing approaches to find out the multidimensional correlation functions. Mixture design is one of the most important statistical techniques used to optimize the ingredients found in the formula considering alternative ingredients. For this, mixture design and RSM can be combined to determine the effects of extraction processing factors. So, combined design was used for obtaining many detailed information about relationship between many independent and dependent factors at specified conditions (Arabameri et al., 2019; Ghelichkhani et al., 2019; Icyer et al., 2016; Rafiei Nazari et al., 2018).

There have been a few reports on the extraction of functional substances from quinoa and barley malt by using superheated water extraction for production of functional beverage. The objective of this study was to support commercial application of quinoa to produce a new functional beverage and to evaluate the potential of SWE as environmentally friendly method for the production of quinoa–barley malt extract. Furthermore, the present study aimed at investigating the effect of SWE parameters including extraction time, particle size, and solid–liquid ratio on yield extraction, and optimized these condition by using D‐optimal combined design.

## 2 MATERIALS AND METHODS

### 2.1 Materials

Quinoa seeds of Sajama variety were obtained from The Karaj Seed and Plant Improvement Institute, Karaj, Iran, in September 2018. Barley malt was purchased from Beh malt Co., and all chemicals used were of reagent grade obtained from Sigma and Merck.

### 2.2 Sample preparation and quinoa/barley malt extraction

Quinoa seeds were separated from impurities (leaves, stones, etc.) and soaked in water for 24 hr and washed thoroughly to remove saponins (foamless) and then dried in ambient temperature. After that, quinoa seeds were ground into powder by an electric grinder (IKA 1603600M 20 Universal Mill, 230V). The milled quinoa flour and barley malt flour were defatted with hexane as solvent in a ratio of 1:5 among 24 hr with the aid of a fattened shaker (Fisher Scientific Ltd, cat. no.14‐285‐729). Afterward, the fat‐free flours were placed for 24 hr in an oven at 40°C, to isolate the residues of solvent, and to obtain good powdered flours. Then all the flours were sieved, using different mesh size sieves (mesh N° (ASTM E11) 40, sieve size 420 µm and No. (ASTM E11) 25, sieve size 710 µm, Sarv Azma Co). Each sample was stored at −18°C until use. Superheated water extraction was performed using Synth wave apparatus (Milestone) with some modification. Quinoa/barley malt were mixed with tap water, the mixture was stirred by an industrial blender at room temperature and 700 rpm for 15 min until a homogeneous mixture was obtained. Subsequently, the substrates were subjected to superheated water extraction apparatus with time–temperature treatments (130°C for 15–45 min). (Alboofetileh et al., 2019).

Total solid was measured and analyzed by drying the sample at 102 ± 2°C to constant weight. Carbohydrate, ash, crude fiber, protein and fat content were determined by standard Association of Official Agricultural Chemists AOAC methods—2005 (AOAC International, 2007). Protein content was determined through nitrogen content estimation by the method of Kjeldahl using a factor of 6.25. pH was measured using a 744‐pH meter Metrohm model (Metrohm) at 20°C. Total carbohydrate content was calculated by subtracting the percentage sum of moisture, protein, fat, crude fiber, and ash from 100%. Turbidity of samples was measured using a turbidometer (2100N Turbidimeter, HACH, CO) and reported in terms of nephalometric turbidity units (NTU). All the analyses were done in triplicate.

Samples were analyzed for total starch using the microwave‐assisted sonication/iodometric USDA Research method (ICUMSA GS1‐16) (Eggleston & Triplett, 2017).

### 2.3 Experimental design and statistical analysis

Statistical analysis was performed by Design Expert version 11.1.2 (Stat‐Ease Inc.). A combination of mixture design and surface response method was applied to evaluate the relationship between independent and dependent variables and to develop a statistical model. Quinoa flour (A) and barley malt flour (B) were selected as the mixture design factors had values from 10% to 30% and 70 to 90%, actual values, respectively. In order to design surface response method, the time (C) ranged from 15–45 min and solid–water ratio (D) ranged from 10:100(0.1), 15:100(0.15), and 20:100(0.2) were selected as processing factors and particle size (E) type (*F* = 420 µm and *G* = 710 µm) was selected as categorical factor. Actual and coded independent variables at various levels are shown in Table 1.

**TABLE 1:** Actual and coded independent variables in the D‐optimal combined design

Name	Symbol	Units	Type	variable level		
				−1	0	+1
Quinoa	*A* (*x*_1_)	%	Mixture	10	15	30
Barley malt	*B* (*x*_2_)	%	Mixture	70	80	90
Time	*C* (*x*_3_)	minutes	Numeric	15	30	45
Solid–water ratio	*D* (*x*_4_)	—	Numeric	0.10	0.15	0.20
Particle size	*E* (*x*_5_)	µm	Categoric	*F*(420)		G(710)

A mixture design is a special response surface experiment in which the parameters are the components of the response and mixture and is a function of the proportions of each component (Santafé‐Moros et al., 2005).

The canonical form of the full quadratic model is shown in Equation (1):

(1) $$Y=\sum_{i=1}^{q}\beta_{i}X_{i}+\sum_{i<j}^{q}\sum \beta_{\mathrm{ij}}X_{i}X_{j}$$

where Y1 (protein), Y2 (carbohydrate), Y3 (turbidity), and Y4 (pH) are the predicted response; $\beta_{i}$ is a linear coefficient, and $\beta_{\mathrm{ij}}$ is a quadratic coefficient. $\beta_{i}X_{i}$ represents the linear blending portion, and the parameter $\beta_{\mathrm{ij}}X_{i}X_{j}$ represents the excess response over the linear model due to the interaction between two components, and this effect is often called antagonism ($\beta_{\mathrm{ij}}<0$) or synergism ($\beta_{\mathrm{ij}}>0$) (Moreira et al., 2007; Santafé‐Moros et al., 2005).

RSM was used for modeling of the processing factors. In this method, the statistical data indicating the correlation between independent factors and the response were adjusted to fit the second‐order polynomial equation in Equation (2) for RSM:

(2) $$Y=\beta_{0}+\sum_{j=1}^{k}\beta_{j}X_{j}+\sum_{i=1}^{k}\beta_{\mathrm{jj}}X_{j}^{2}+\sum_{i=1}^{j‐1}\sum_{j=i+1}^{k}\beta_{\mathrm{ij}}X_{i}X_{j}+\varepsilon$$

in Equation (2); *i* and *j* are the linear quadratic coefficient; *X_i_* and *X_j_* are the encoded independent variables; k is the number of studied factors optimized; $\beta_{0}$ is the constant (intercept); *β_i_* the linear coefficient; $\beta_{j}$, $\beta_{\mathrm{jj}},\;\text{and}\;\beta_{\mathrm{ij}}$ are interaction coefficient of linear, quadratic, and second‐order terms, respectively; *X_i_* and *X_j_* are independent variables and ε is the error. Statistical significance of the model parameters was set at the 5% (*p*‐value < .05).

## 3 RESULTS AND DISCUSSION

### 3.1 Goodness of fit models

Statistical analysis parameters of the Fisher test value (*F*‐value), *p*‐value of model, coefficient of determination (*R* ^2^), the coefficients of determination (*R* ^2^‐adj), predicted *R* ^2^, *p*‐value of lack of fit(LOF), adequate precision, and CV% obtained from the analysis of variance (ANOVA) were used for evaluation of the goodness of fit in models. Fitted polynomials models were applied for optimization of extraction conditions. Statistical significance of the model parameters was set at the 5% (*p*‐value < .05). Table 2 reports the corresponding 37‐run D‐optimal combined experimental plan and obtained responses.

**TABLE 2:** 37‐run D‐optimal combined experimental plan and obtained responses

Run	Component 1	Component 2	Factor 3	Factor 4	Factor 5	Response 1	Response 2	Response 3	Response 4
	A: Quinoa flour	B: Malt barley flour	C: Time	D: Solid–Water	E: Particle size	Protein	Carbohydrate	Turbidity	pH
1	30.000	70.000	45.00	0.20	Particle size G	1.26	11.09	395	5.43
2	10.000	90.000	15.00	0.10	Particle size F	0.52	6.93	146	5.74
3	20.000	80.000	45.00	0.15	Particle size F	0.94	9.35	230	5.37
4	20.000	80.000	30.00	0.20	Particle size F	0.75	10.3	314	5.51
5	20.000	80.000	15.00	0.10	Particle size F	0.55	7.39	158	5.77
6	10.000	90.000	15.00	0.15	Particle size G	0.53	8.37	174	5.76
7	20.000	80.000	15.00	0.20	Particle size G	0.59	10.01	272	5.78
8	30.000	70.000	45.00	0.20	Particle size G	1.25	11.09	395	5.45
9	25.000	75.000	45.00	0.10	Particle size F	0.95	8.35	201	5.4
10	30.000	70.000	45.00	0.10	Particle size G	0.99	8.63	177	5.41
11	10.000	90.000	15.00	0.20	Particle size F	0.54	9.63	219	5.76
12	30.000	70.000	15.00	0.20	Particle size G	0.71	10.31	346	5.83
13	30.000	70.000	15.00	0.20	Particle size F	0.72	10.31	281	5.84
14	10.000	90.000	30.00	0.15	Particle size F	0.68	8.56	221	5.46
15	20.000	80.000	45.00	0.10	Particle size G	0.93	8.1	170	5.34
16	30.000	70.000	15.00	0.10	Particle size F	0.67	7.85	170	5.82
17	20.000	80.000	15.00	0.10	Particle size G	0.53	7.43	185	5.77
18	10.000	90.000	15.00	0.20	Particle size F	0.54	9.63	219	5.75
19	10.000	90.000	15.00	0.10	Particle size F	0.52	6.93	146	5.74
20	10.000	90.000	45.00	0.10	Particle size G	0.8	7.57	164	5.31
21	20.000	80.000	45.00	0.20	Particle size G	0.95	10.68	315	5.39
22	10.000	90.000	45.00	0.20	Particle size F	0.89	10.18	282	5.33
23	25.000	75.000	45.00	0.15	Particle size G	0.97	9.62	235	5.4
24	10.000	90.000	45.00	0.10	Particle size F	0.81	7.49	144	5.32
25	30.000	70.000	15.00	0.10	Particle size G	0.65	7.85	178	5.8
26	20.000	80.000	30.00	0.15	Particle size G	0.72	9.06	232	5.5
27	10.000	90.000	45.00	0.20	Particle size G	0.86	10.27	235	5.33
28	30.000	70.000	45.00	0.15	Particle size F	1.11	9.86	268	5.42
29	20.000	80.000	15.00	0.20	Particle size F	0.61	9.97	250	5.79
30	25.000	75.000	30.00	0.10	Particle size G	0.75	8	190	5.54
31	30.000	70.000	15.00	0.20	Particle size G	0.7	10.76	372	5.83
32	15.000	85.000	30.00	0.10	Particle size F	0.7	7.65	178	5.48
33	30.000	70.000	45.00	0.15	Particle size F	1.05	10.29	279	5.42
34	10.000	90.000	30.00	0.20	Particle size G	0.69	10.27	274	5.47
35	30.000	70.000	30.00	0.15	Particle size F	0.79	8.79	207	5.57
36	30.000	70.000	15.00	0.10	Particle size G	0.64	7.58	173	5.81
37	30.000	70.000	30.00	0.10	Particle size F	0.78	8.16	192	5.56

Protein content response was fitted to Quadratic x Quadratic model, which mean that both the mixture and process factor fit to quadratic models. According to Table 3, the *p*‐value for the model shows that the models are significant at less than a .05 level. In this case, A, B, AB, AC, AD, BC, BD, CD, ABD, ACD, AC^2^, AD^2^, and ABCD have significant effect on protein response.

**TABLE 3:** Parameter estimates and analysis of variance for responses model*

Response	*F*‐value of model	*p*‐value of model	*R* ^2^	Adjusted *r* ^2^	Predicted *r* ^2^	*p*‐value lack of fit	Adequate precision	C.V. %
Protein	455.97	<.0001*	.9961	.9940	.9904	.77	77.13	235
Carbohydrate	122.04	<.0001*	.9909	.9828	.9612	.79	36.00	1.84
Turbidity	218.03	<.0001*	.9949	.9903	.9755	.6059	53.35	1.44
pH	85,750	<.0001*	.9987	.9975	.9954	.1827	80.56	0.168

* *p* < .05 is significant.

Carbohydrate was fitted to Linear x Quadratic model. Based on the ANOVA analysis, model *p*‐values < .05 indicate model terms are significant. The final equation, expressed in terms of the coded factors, is shown in Table 5. For this response, A, B, AC, AD, BC, BD, AC^2^, and BC^2^ are significant model terms.

Turbidity was fitted to Linear x Quadratic model. According to the ANOVA analysis shown in Table 3, the model is significant as model *p*‐value less than a 0.05. In this case, A, B, AC, AD, BC, BD, CD, ACD, ACE, ADE, BCE, BDE, AC^2^, AD^2^, BC^2^, and BD^2^ are significant model terms.

The pH was fitted to Linear x Quadratic model. *p*‐values < .05 indicate model terms are significant. In this case, A, B, AC, AD, BC, BD, AC^2^, and BC^2^ are significant model terms.

*p*‐value lower than .0001 was found, demonstrating the high significance of the regression model and can be used to optimize the variables. As seen from Table 3, *F*‐value of model was 455.97 for protein, 122.04 for carbohydrate, 218.03 for turbidity, and 857.50 for pH.

The quality of fitting to the selected models was confirmed by the attained high values for the coefficient of determination obtained for protein content (*R* ^2^ = .9961), carbohydrate content (*R* ^2^ = .9909), turbidity (*R* ^2^ = .9949), and pH (*R* ^2^ = .9987) are presented in Table 3. High *R* ^2^ value shows higher correlation between the independent variables and the responses.

Adjusted coefficient (Adj‐*R* ^2^) was for protein content (.9916), carbohydrate content (.9828), turbidity (.9903) and pH (.9975). The value of the adjusted coefficient of determination (*R* ^2^ Adj) also indicates an excellent correlation, supporting that this model explains the experimental results adequately.

As shown in Table 3, all of the predicted *R* ^2^ of responses were above of .80. High values of predicted *R* ^2^ represent a high degree of correlation between the experimental and predicted values.

According to Table 3, for all responses *p*‐values of LOF were higher than .05. Non‐significant lack of fit of responses is good for models. Adequate precision measures the signal‐to‐noise ratio. A ratio greater than 4 is desirable (Wang et al., 2018). According to Table 3, adequate precision of the responses ranged from 36.00 to 80.56. These results imply the validity of models.

As seen from Table 3, coefficient of variation (CV %) of model for protein content, carbohydrate content, turbidity, and pH was 2.35, 1.84, 1.44, and 0.16, respectively. The CV% describes the extent to which the data were dispersed. Since CV is a measure expressing standard deviation as a percentage of the mean, the small values of CV give better reproducibility (Hou et al., 2019).

The predicted models for the protein content (Y1), carbohydrate content (Y2), turbidity (Y3), and pH (Y4) of the extracts are presented in Table 4 by the following equations, respectively. The following regression equations predict the value of each response variable when the independent factors are varied; positive sign in front of the terms indicates synergistic effect, whereas negative sign indicates antagonistic effect.

**TABLE 4:** Final equation of responses

Response	Equations
Protein	0.806859 * A + 0.683919 * B − 0.139351 * AB + 0.219979 * AC + 0.0808641 * AD + 0.15535 * BC + 0.0200393 * BD + 0.0122301 * CD − 0.0100473 * ABC − 0.122535 * ABD + 0.0409079 * ACD^2^ + 0 * BCD + 0.0461823 * AC^2^ + 0.0478067 * AD^2^−0.162343 * ABCD (1)
Carbohydrate	9.10533 * A + 8.7647 * B + 0.441027 * AC + 1.2354 * AD + −0.0161247 * AE + 0.246551 * BC + 1.32635 * BD + 0.0829342 * BE + 0.0279301 * CD − 0.0958262 * ACD + −0.056747 * ACE + 0.0628723 * ADE + 0 * BCD − 0.0158739 * BCE − 0.0361824 * BDE + 0.344936 * AC^2^ + 0.0771517 * AD^2^ − 0.288276 * BC^2^ + 0.144163 * BD^2^ (2)
Turbidity	14.9453 * A + 14.9914 * B + 0.774874 * AC + 2.55898 * AD + 0.13833 * AE + 0.275434 * BC + 1.33519 * BD + 0.0825583 * BE + 0.554096 * CD − 0.325745 * ACD − 0.500184 * ACE + 0.470998 * ADE + 0 * BCD − 0.186029 * BCE − 0.598934 * BDE + 0.367529 * AC^2^ + 0.955265 * AD^2^ − 1.67316 * BC^2^ + 0.649508 * BD^2^ (3)
pH	5.56342 * A + 5.45909 * B − 0.197291 * AC + 0.0111604 * AD − 0.00295748 * AE − 0.212992 * BC + 0.00922283 * BD − 0.000727889 * BE − 0.000194327 * CD + 0.00193814 * ACD + 0.00172143 * ACE + 0.00173795 * ADE + 0 * BCD − 0.00210217 * BCE + 0.00368023 * BDE + 0.052932 * AC^2^ + 0.0074094 * AD^2^ + 0.0808962 * BC^2^ − 0.00595566 * BD^2^ (4)

**TABLE 5:** The proximate composition of quinoa and barley malt flour (g/100 g d. w. basis)

The proximate composition	Quinoa flour	Barley malt flour
Protein	15.76 ± 0.2	11.13 ± 0.2
Carbohydrates	68.71 ± 0.5	69.8 ± 0.4
Starch	54.4 ± 0.2	60.24 ± 0.5
Fat	5.32 ± 0.3	4.46 ± 0.09
Ash	2.84 ± 0.2	2.04 ± 0.08
pH	6.40 ± 0.08	6.07 ± 0.06

Note

Results are given as mean ± *SD*.

### 3.2 Response 1, protein content

**FIGURE 1:**
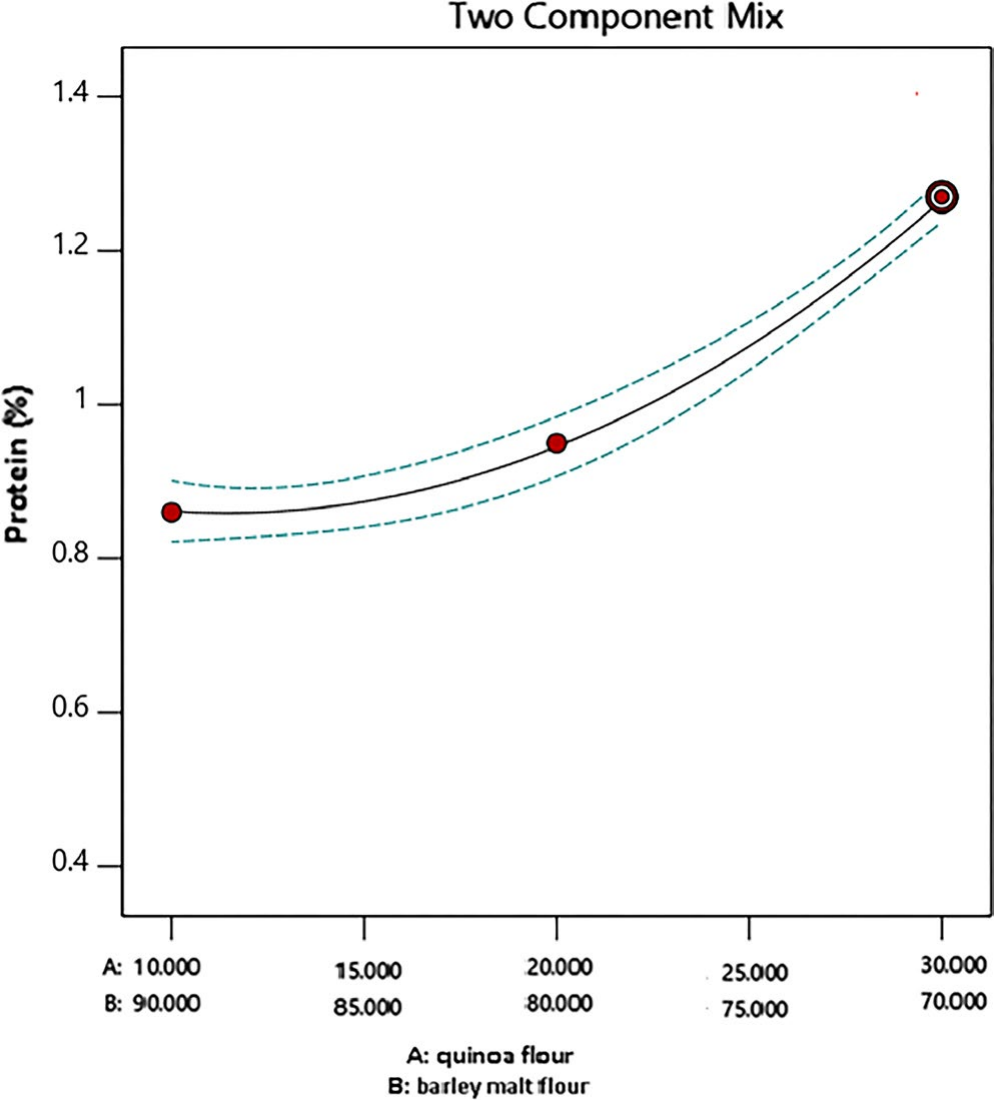
The effect of quinoa/barley malt flour on protein (%)

Figure 1 shows the effect of the quinoa/barley malt ratio on protein content (%) values according to the model analysis. This figure shows that the minimum amount of protein was produced when this ratio is 10/90. As tabulated in Table 5, the protein content of quinoa flour (15.76 g/100 g dry basis, d.b.) was higher than barley malt (11.13 g/100 g d.b.). Generally, quinoa seeds have a higher nutritional value, in comparison with most cereals. The quinoa protein content varies from 14 to 20 (g/100 g d.b.) (Matiacevich et al., 2006) which on average is higher than common cereals such as rice, wheat, and barley malt (Valencia‐Chamorro, 2003). As shown in Figure 1 by increasing the quinoa flour in mixture component, the protein content increased and the maximum values of protein from this mixture obtained when the quinoa/barley malt ratio was 30/70.

**FIGURE 2:**
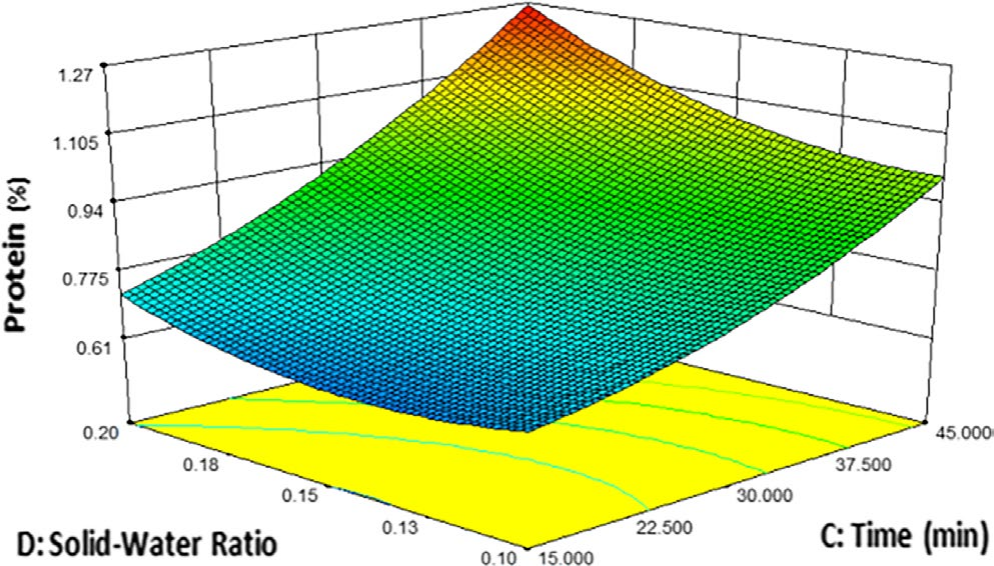
The effect of time and solid–water on protein (%)

The test results as reflected in Figure 2 show the interaction between solid–water ratio and time (CD) on the protein content while the other independent variable was constant at 20% quinoa, 80% barley malt, and average of particle size F and G. From Figure 2, it can be concluded that the percentage of protein increased with increasing extraction time and solid–water ratio as processing factor. Maximum protein was extracted when both factors were at high level (Time = 45min and solid–water ratio = 0.2). It means both factors have a positive effect on the protein content.

### 3.3 Response 2, carbohydrate content

**FIGURE 3:**
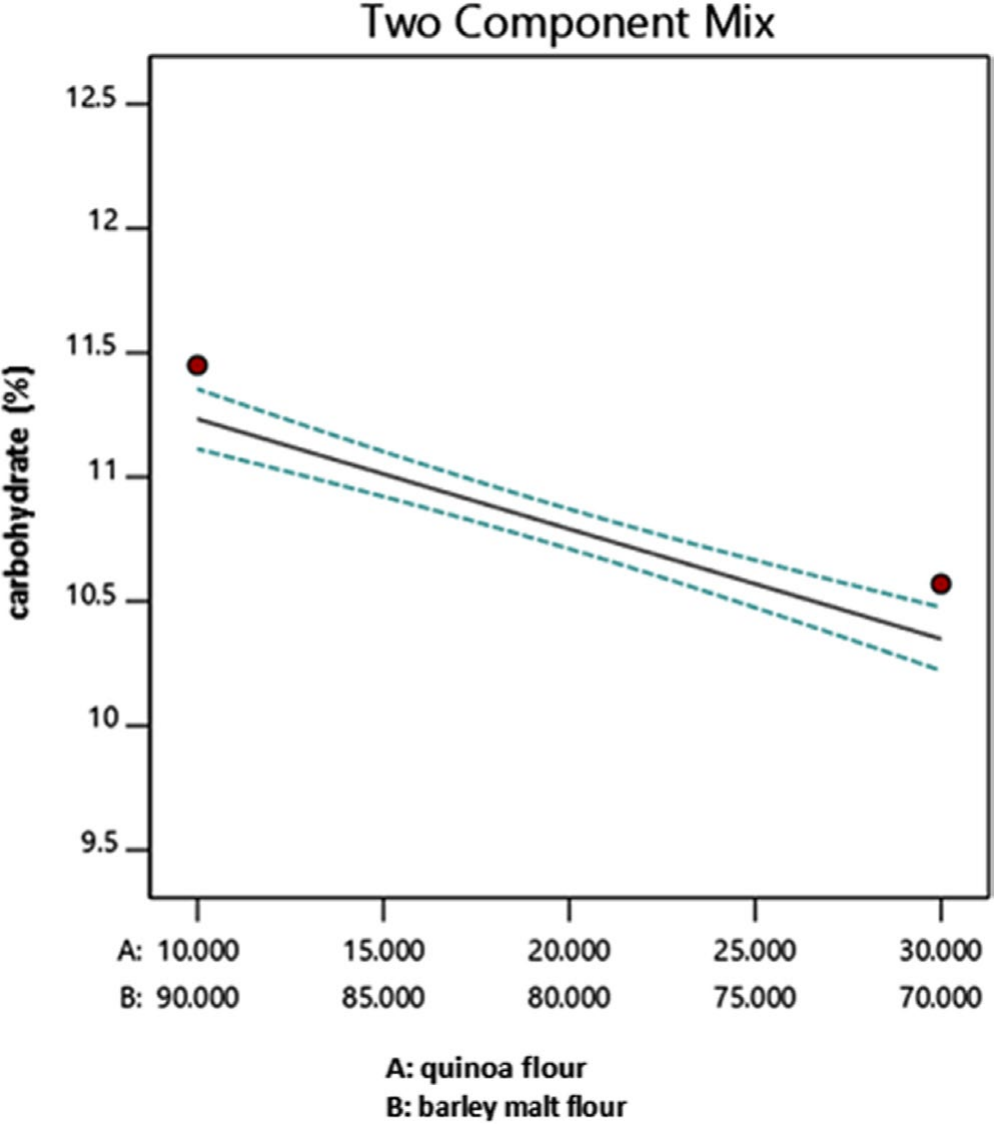
The effect of quinoa/barley malt flour on carbohydrate (%) (both particle size F and G)

Figure 3 shows the effect of the quinoa/barley malt ratio on carbohydrate content (%) values according to the selected model. The results showed that carbohydrate content decreased as quinoa/barley malt ratio increases. According to Table 5, the carbohydrate content of barley malt is higher than quinoa, and beside the carbohydrate content (%) test results, the increase in carbohydrate content was obvious for the samples with higher barley malt content during the extraction processing. The maximum values of carbohydrate obtained when the quinoa/barley malt ratio is 10/90.

As shown in Table 5, the starch of quinoa and barley malt is 54.4. The study of Pulvento et al in 2012 demonstrates that available carbohydrates in quinoa not only include starch but also other components; however, more researches on the nature of non‐starch available carbohydrates are needed (Pulvento et al., 2012). According to this table, starch constitutes 60.24% of barley malt.

**FIGURE 4:**
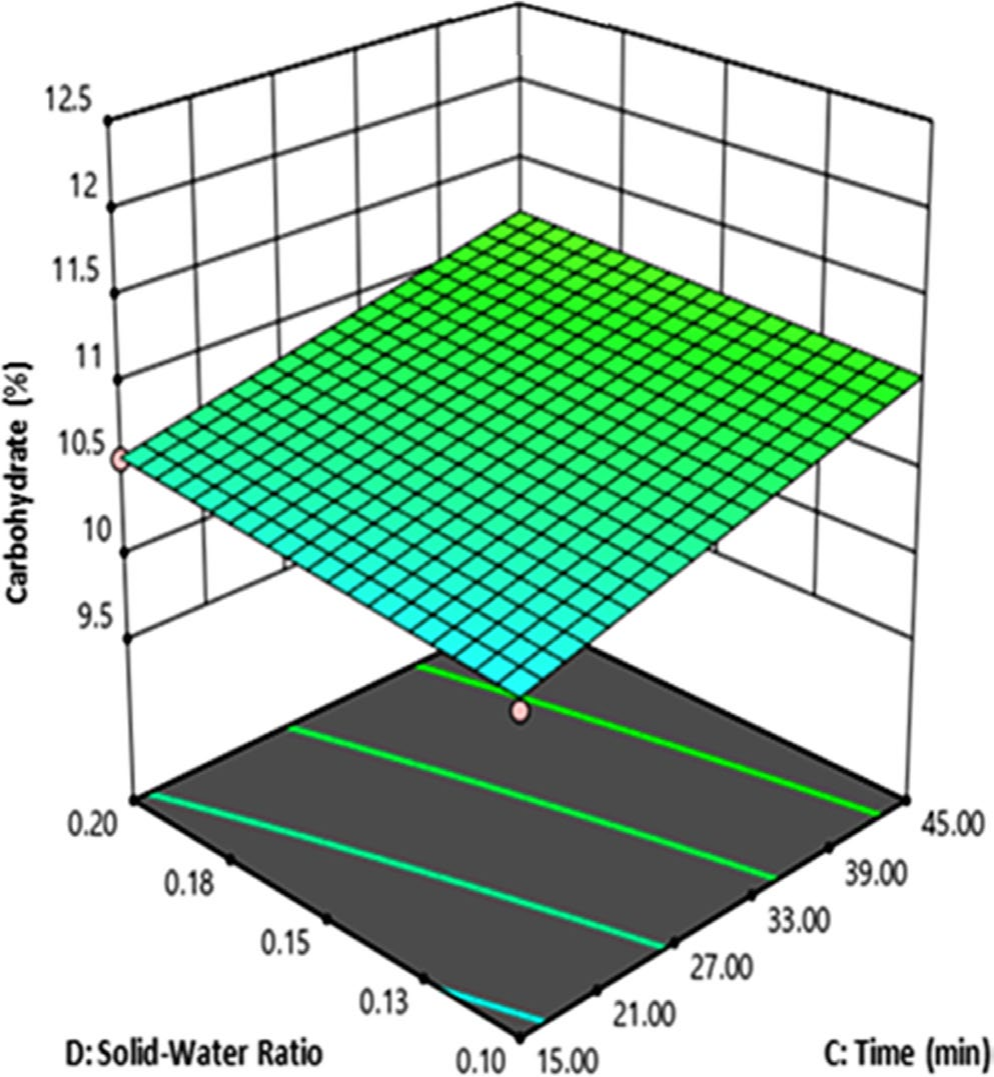
The effect of time and solid–water on carbohydrate (%) (both particle size F and G)

Under hydrothermal conditions such as subcritical water extraction method, carbohydrates undergo rapid hydrolysis to monosaccharides, which are then further degraded. Starch and hemicelluloses are hydrolyzed much faster than cellulose, which in contrast to the former two has a mainly crystalline structure (Toor et al., 2011). The degradation of carbohydrates to glucose and other saccharides in sub‐ and supercritical water has been reviewed by several authors (Behrendt et al., 2008; Bröll et al., 1999; Yu et al., 2008). Figure 4 shows the effect of extraction time (C) and solid–water ratio (D) on this response at 20% quinoa flour, 80% barley malt, and average of particle size F and G. According to this figure, when both the solid–water ratio and extraction time increase, the carbohydrate content increased. Maximum carbohydrate yielded when solid–water ratio was at high level (0.2) and time was at the highest level (45 min).

### 3.4 Response 3, Turbidity

**FIGURE 5:**
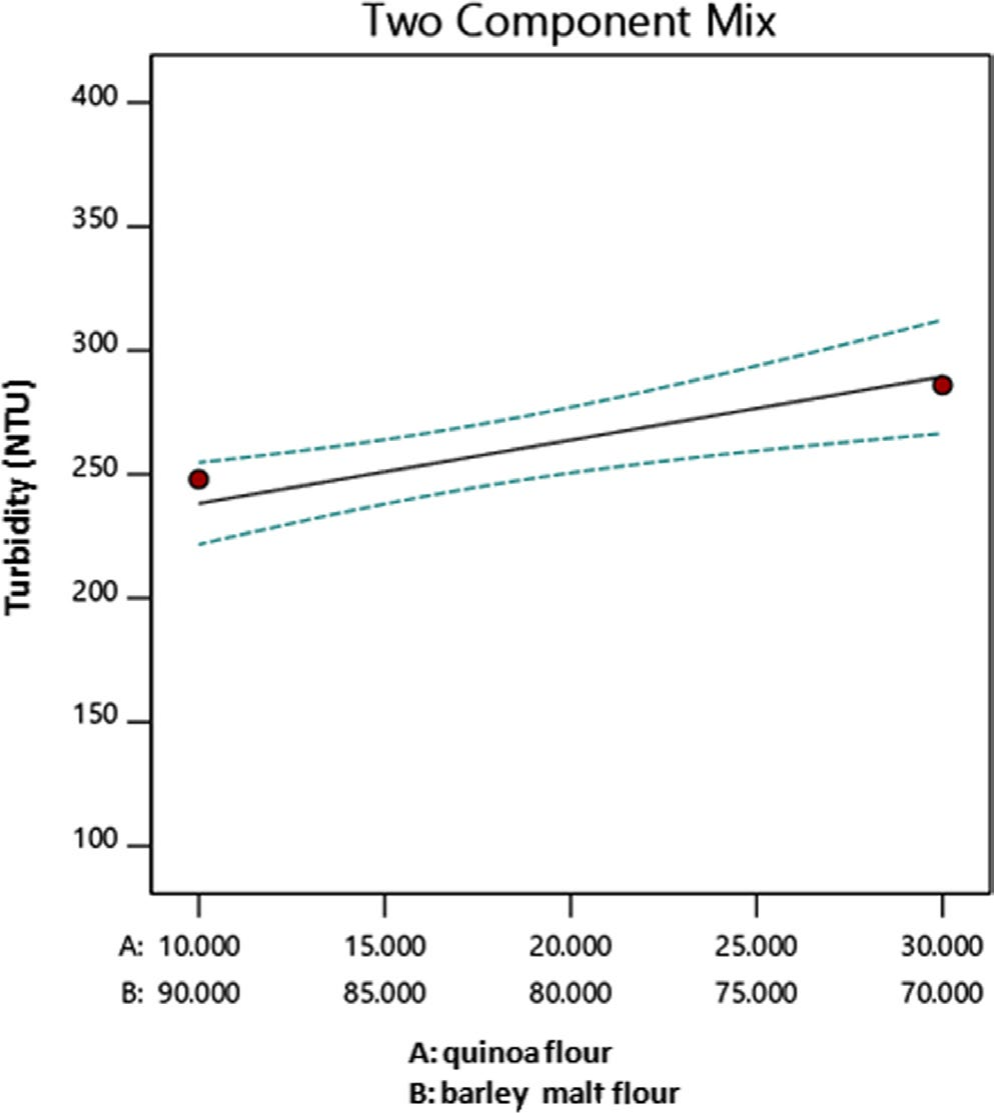
The effect of quinoa–barley malt flour on turbidity (NTU)

The effect of the quinoa/barley malt ratio on turbidity (NTU) at extraction time 30 min, solid–water ratio 0.15, and particle size G is depicted in Figure 5. It can be observed that an increase in the quinoa content and decrease in barley malt in formulation of extract linearly increases the turbidity (NTU) of sample, the minimum amount of turbidity (NTU) is obtained when quinoa/barley malt ratio is 10/90, and turbidity is at maximum when this ratio is 30/70.

Protein is one of the important factor that affects turbidity in beverages, proteins have been associated with hazes in beer, red and white wine, apple, grape juice, and kiwifruit juice (Siebert & Lynn, 2000). According to Table 4, the protein content of quinoa is higher than barley malt and by increasing the amount of protein in extract the turbidity was increased, although the other factors such as polysaccharide and polyphenol have been implicated in hazes of many beverage. A number of polysaccharides have been associated with beverage such as beer hazes or flocs, which include beta‐glucans, starch, and mannan (Stounbjerg et al., 2018). Thus beta‐glucan and starch of barley malt and the starch of quinoa may influence on turbidity of quinoa–barley malt extract. One should not neglect the interaction of this polysaccharide with protein of the extract to alter the turbidity as well. However, several studies have been carried out on interaction of protein–polysaccharide, (Duran et al., 2018; Sommer et al., 2019; Tavernier et al., 2017), but polysaccharide–protein interactions of quinoa in beverage have not been widely investigated yet. The knowledge of these properties can contribute to the understanding of the effect of polysaccharide–protein interaction of quinoa on turbidity of beverages and support commercial application of quinoa to produce a new functional beverage.

**FIGURE 6:**
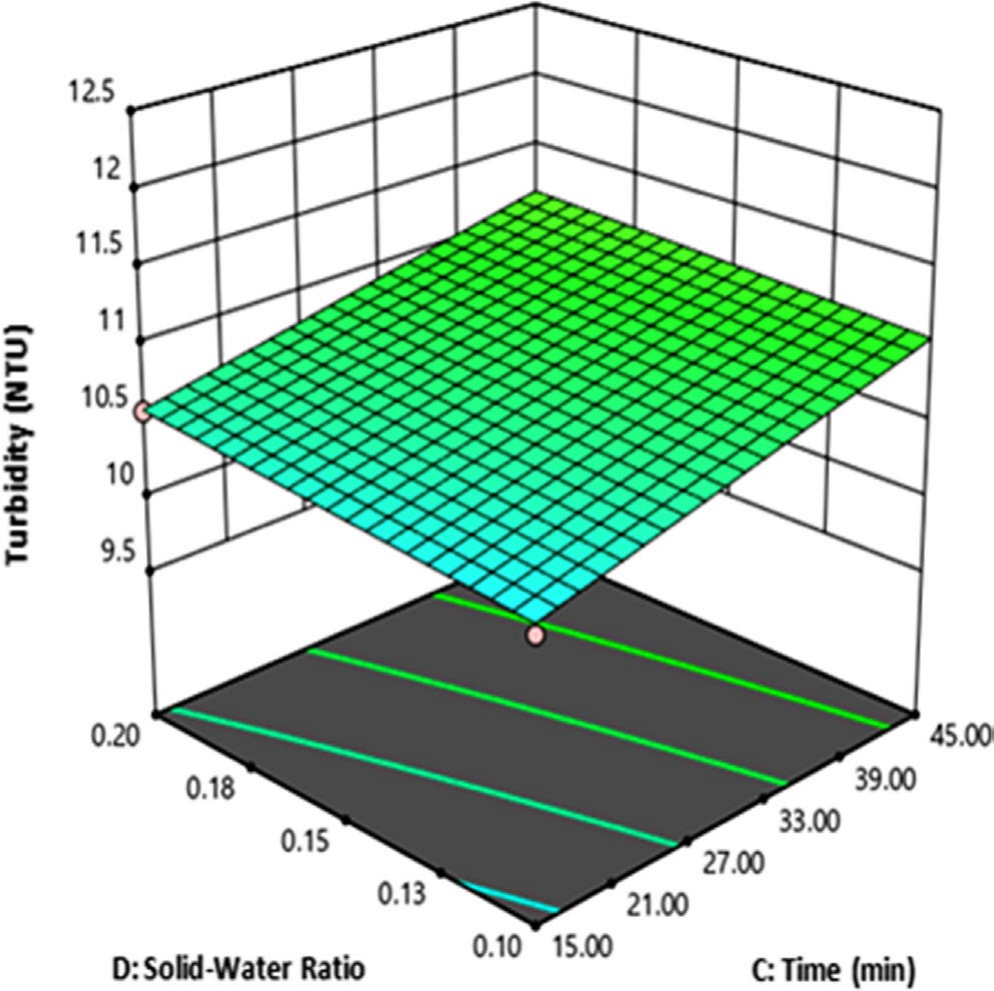
The effect of time and solid–water on turbidity

Surface plot (3D) was constructed as shown in Figure 6. The 3D surface plots depict the interaction of two independent variables (C: Time and D: solid–water ratio) on turbidity response while the other independent variables were constant (at 20% quinoa, 80% barley malt in average of particle size F and G). The results in Figure 6 indicate that maximum turbidity was produced when both factors were at high level (Time = 45min and solid–water ratio = 0.2). It means both factors have a positive effect on the turbidity.

### 3.5 Response 4, pH content

**FIGURE 7:**
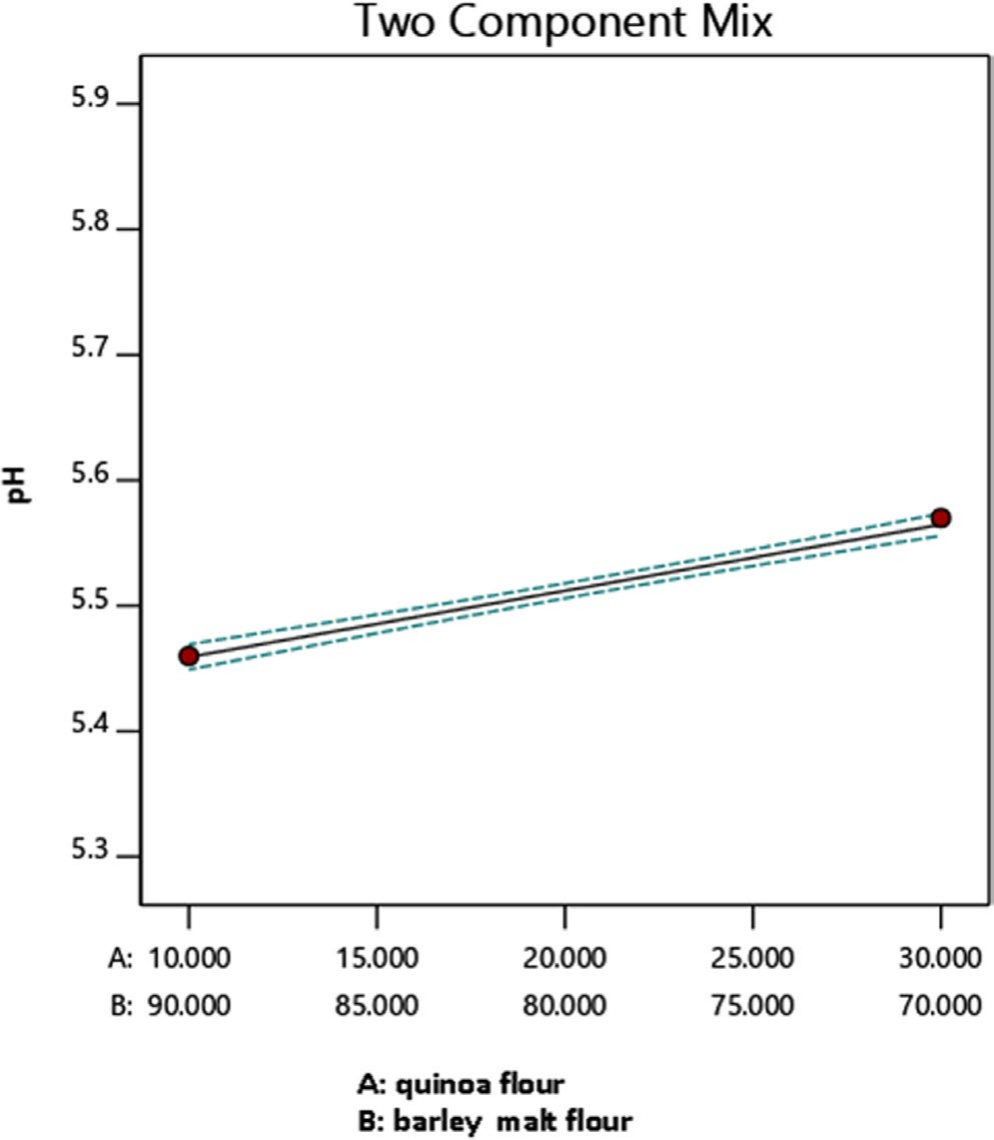
The effect of quinoa–barley malt flour on pH

Figure 7 illustrates the effect of quinoa/barley malt ratio on pH response at 20% quinoa, 80% barley malt in average of particle size F and G. The test results indicate that by increasing the quinoa/barley malt ratio, pH increased. According to Table 5, the pH of quinoa is 6.40 and higher than the pH of barley malt that is 6.07. Results presented in Figure 7 show that the minimum of this response is obtained when quinoa/barley malt ratio is 10/90 and the maximum of pH was produced when this ratio was 30/70.

**FIGURE 8:**
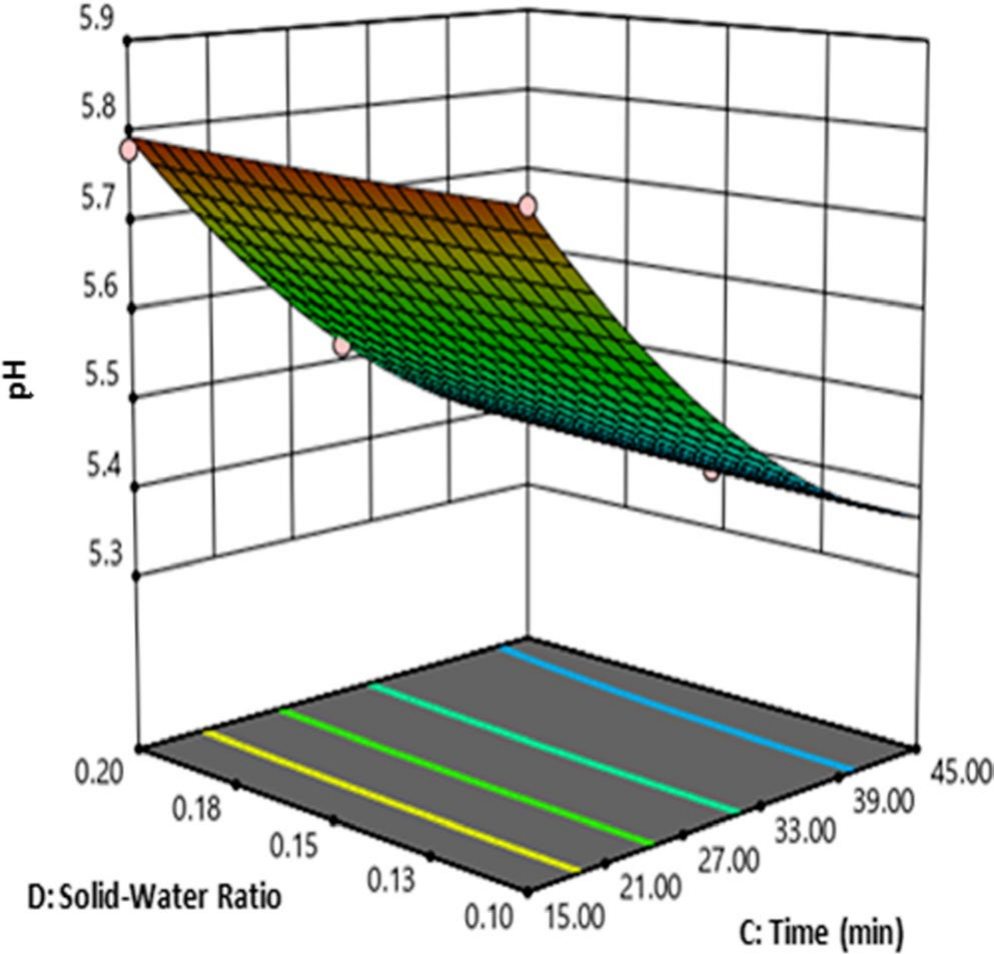
The effect of time and solid–water on pH

The response surface is plotted to study the interaction between concentration and time (CD) on the pH (Figure 8). The test results indicated that by increasing the extraction time pH decreased, by increasing the solid–water ratio pH increased. The minimum pH was obtained when extraction time and solid–water ratio are at high level (time = 45 min and solid–water ratio = 0.2). Decomposition of biomass, carbohydrates, and amino acids under subcritical water condition produces acidic component such as organic acid. Due to the formation of water‐soluble organic acids, the pH of the treatment medium may be decreased (Lamoolphak et al., 2006; Pourali et al., 2009; Salak Asghari & Yoshida, 2006).

## 4 OPTIMIZATION OF EXTRACTION CONDITIONS

The main purpose of this study was to determine the optimum extraction conditions time (min), solid–water ratio and particle size of quinoa–barley malt flours. Derringer's desirability functions were applied for the selection of optimum extraction conditions for production of fermented probiotic beverages in next step. Overall Derringer's desirability function was established for the criteria that maximum protein and carbohydrate content and minimum turbidity and pH (Granato & Ares, 2014). It required high amount of protein and carbohydrate to have an optimum nutritional source for probiotic bacteria, used in the functional beverage. Moreover, for better growth of probiotics in acidic environment, a possible minimum pH is selected. For a clear fermented beverage, we considered a minimum turbidity.

So we selected maximum protein, maximum carbohydrate, minimum turbidity, and minimum pH to optimize procedure by design expert software.

The optimum amount of quinoa flour, barley malt flour, solid–water ratio, time, and particle size was 30%, 70%, 0.2, 45 min, and *F* = 420 µm, respectively. The overall desirability for the optimized condition was equal to 0.996 indicating the chosen solution stood in a good place.

In order to validate the results of optimization, three replicates of experiments for each response were performed under the optimal conditions. The comparison of experimental results with the predicted results under optimal condition is carried out by Paired‐*t* test. The results of validity evaluation of optimal conditions are summarized in Table 6. As shown, insignificant differences between experimental results and predicted results imply on validity of optimal conditions.

**TABLE 6:** Results of validity evaluation of optimal condition

Response	Predicted value	Experimental value	*p*‐value (paired‐*t* test)
Protein	1.279 ± 0.04	1.256 ± 0.015	.52
Carbohydrate	11.09 ± 0.115	11.14 ± 0.095	.061
Turbidity	388.107 ± 26.16	398.333 ± 20.207	.738
pH	5.438 ± 0.013	5.433 ± 0.035	.771

## 5 CONCLUSIONS

This study was aimed to optimize the production of quinoa–barley malt extract by using superheated water extraction method. The designed model results proved that D‐optimal combined design methodology is a very effective method to reach very accurate results in the design of superheated water extraction condition. Results from responses (protein, carbohydrate, turbidity, and pH) demonstrated the regression models were accurate within the set of values of the included variables. The result indicated that increase in quinoa/barley malt ratio increase the protein content whereas the carbohydrate values linearly decrease. Quinoa/barley malt ratio also affected on physicochemical properties such as turbidity and pH. Both of these parameters increased when quinoa/barley malt ratio increased. In this study, the protein and carbohydrate value was affected by superheated water extraction parameters. By increasing solid/water ratio and extraction time, protein and carbohydrate content increased. The optimal SWE conditions at quinoa 30%, barley malt 70%, solid–water ratio 0.2% at 45 min, and particle size F are obtained. It is necessary to select the suitable extraction technique and optimize the extraction parameters in order to obtain the highest yield of valuable material. In conclusion, although SWE appears to be a useful extraction method for production of valuable materials from cereal and pseudocereal, this method has only been used at pre‐commercial scale and more investigation is required to study the quantity, quality, and stability of the extracted valuable materials to scale it up for industrial means.

## Data Availability

Data are openly available in a public repository that does not issue DOIs, as mentioned in Table 2.
